# On learning agent-based models from data

**DOI:** 10.1038/s41598-023-35536-3

**Published:** 2023-06-07

**Authors:** Corrado Monti, Marco Pangallo, Gianmarco De Francisci Morales, Francesco Bonchi

**Affiliations:** CENTAI, Turin, Italy

**Keywords:** Computer science, Computational science

## Abstract

Agent-Based Models (ABMs) are used in several fields to study the evolution of complex systems from micro-level assumptions. However, a significant drawback of ABMs is their inability to estimate agent-specific (or “micro”) variables, which hinders their ability to make accurate predictions using micro-level data. In this paper, we propose a protocol to learn the latent micro-variables of an ABM from data. We begin by translating an ABM into a probabilistic model characterized by a computationally tractable likelihood. Next, we use a gradient-based expectation maximization algorithm to maximize the likelihood of the latent variables. We showcase the efficacy of our protocol on an ABM of the housing market, where agents with different incomes bid higher prices to live in high-income neighborhoods. Our protocol produces accurate estimates of the latent variables while preserving the general behavior of the ABM. Moreover, our estimates substantially improve the out-of-sample forecasting capabilities of the ABM compared to simpler heuristics. Our protocol encourages modelers to articulate assumptions, consider the inferential process, and spot potential identification problems, thus making it a useful alternative to black-box data assimilation methods.

Agent-Based Models (ABMs) are computational models in which autonomous “agents” interact with one another and with their environment, thereby producing aggregate emergent phenomena^1^. ABMs are an extremely successful tool for theory development, that is, to explore the macro-level implications of micro-level assumptions^2^. As Axelrod^3^ said *“whereas the purpose of induction is to find patterns in data [...], the purpose of agent-based modeling is to aid intuition”*. In line with this focus on theory development, the ability of ABMs to match empirical data and make quantitative forecasts—that is, to *learn from data*—has been, so far, limited^4–7^.

At a very high level, all ABMs can be described by the formula

1 $$\begin{aligned} Z_t \sim \mathbb {P}_t \left( Z_t \mid \Theta , Z_{\tau < t} \right) , \end{aligned}$$

where $Z_t$ are the *variables* of interest in the system, $\Theta$ is a set of *parameters*, $\mathbb {P}_t$ is a probability function implicitly defined by model specifications, and *t* is the discrete time index. Typically, $\Theta$ has relatively few components and a fixed dimensionality, is interpretable by a domain scientist, and is the only tuning knob of the model. *Z* is at the same time the state and the output of the model. Each component of *Z* typically refers to an individual agent, which results in high dimensionality. Some of *Z* is *observable*, while the rest is *latent*.

Most of the efforts in learning agent-based models from data have focused on parameter calibration. This task refers to the process of finding a parametrization $\Theta$ that can reproduce some macroscopic characteristic of the data, and it typically boils down to comparing a few summary statistics of aggregate empirical and model-generated variables (e.g., time series)^6,8^. Summary statistics are valuable to focus on the most important characteristics of the data that the modeler wants to explain, but they often have to be chosen arbitrarily, and may hide very different underlying patterns (as in the well-known Anscombe quartet).

Estimating agent-specific (or “micro”) *variables* *Z* is instead not usually considered. We think this is the main obstacle to bringing ABMs closer to data and potentially using them as a forecasting tool. Indeed, if modelers do not correctly initialize latent micro variables and update their values as the simulation progresses, and if the dynamics of observed variables crucially depend on the initialization of latent variables, then model-generated time series are bound to diverge from empirical ones. Additionally, this mismatch implies that model-generated and empirical time series cannot be directly compared to produce a “goodness-of-fit” measure, so one must resort to summary statistics or “stylized facts” to calibrate parameters. The ABM community has recently started to explore data assimilation methods to estimate the latent variables of ABMs^4,9–11^; we explain the relation of this literature to our work in the Discussion.

This paper proposes a general methodology for estimating latent variables in ABMs. Our approach proceeds in three steps:

1. Given an ABM of reference, translate it into a probabilistic model, by simplifying it until a computationally tractable likelihood of the data given the latent variables can be specified.
2. Estimate latent variables at each time step while keeping all past input values fixed (as in online learning). Solidly rooted in probability theory, our approach maximizes the likelihood at each step via expectation-maximization^12^ and gradient descent.
3. Repeat this process over multiple epochs, so that temporal dependencies can be appropriately taken into account.

We showcase our approach by applying it to a housing market ABM specifically designed to study income segregation^13^ (Fig. 1). We use the ABM to generate synthetic data traces that we can use as ground truth (we would not have access to a ground truth for latent variables if we used real-world data traces). We distinguish between observable and latent variables based on how easy it usually is to access the relevant real-world data. The main latent variable in this model is the distribution of agents’ incomes in each neighborhood, which is often not available. We instead assume that we observe neighborhood-level mean prices and the number of transactions over time (these data are usually readily available, see e.g.^14^). We believe this distinction to reflect a common real-world setting in economic models: one might have access to which actions are being performed, but not to the latent state of agents (e.g., where they live, and what is their income). We write the likelihood of prices and of the number of transactions as a function of the household income distribution. Next, we maximize this likelihood, thus estimating the time evolution of the spatial income distribution.

**Figure 1:**
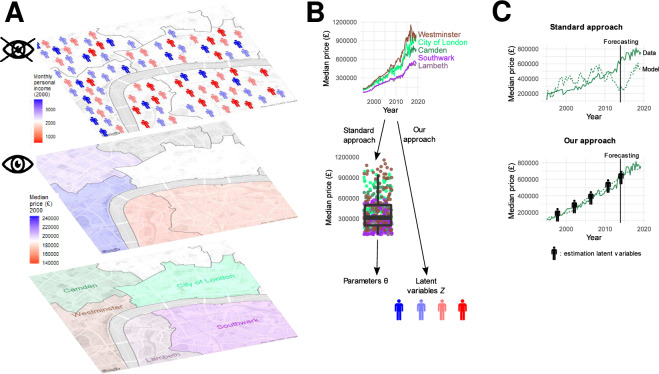
Our approach compared to a standard approach towards calibrating an ABM of the housing market. (**A**) Focusing on the boroughs in the center of London (bottom layer), we consider the yearly average of transaction prices (middle layer) as an example of an observed variable, and the distribution of agent incomes (top layer) as an example of a latent variable. (**B**) For each borough, we observe a time series of transaction prices. In the standard approach to calibration, modelers could typically calibrate some parameters $\Theta$ (such as the probability for inhabitants to put their home on sale) by computing the moments of transaction prices across boroughs and years (as represented through a box plot), and minimizing the distance with the same moments in model-generated time series. In our approach, instead, we are able to calibrate the evolution of latent variables *Z* –in this example, borough-level agent incomes– by exploiting all information contained in time series, rather than reducing this information to specific summary statistics. (**C**) In the model, prices depend on agent incomes. Thus, since in the standard approach agent incomes are not calibrated, model-generated time series are bound to diverge, even if prices are initialized as in the data. With our approach, as we repeatedly estimate incomes, we can make model-generated time series track empirical ones. This makes it feasible to forecast future prices.

In synthetic experiments, we show that our procedure enables learning latent variables accurately: the Pearson correlation between the ground truth and learned traces ranges between 0.5 and 0.9, depending on the latent variable considered. At the same time, we show that an accurate estimation of latent variables empowers out-of-sample forecasting. Compared to other benchmarks that use rules of thumb to initialize the model at the beginning of the forecasting window, our procedure obtains lower Root Mean Squared Error (RMSE) with respect to the ground truth while being more principled. It also highlights potential identification problems, i.e., situations wherein multiple configurations of micro-variables correspond to the global maximum of the likelihood, so that the ground truth configuration cannot be identified.

## Model

We start from the housing market ABM presented by Pangallo et al.^13^—henceforth, the “original ABM”. Our goal is to modify the original ABM until it is possible to write a computationally tractable likelihood of observed variables given latent variables and parameters. If we are able to do so, we say that the modified model is a *learnable* ABM. While writing a tractable likelihood function, we need to preserve the general behavior of the model, as well as its essential causality mechanisms.

In this section, we first give an overview of the original ABM; then, we describe the learnable ABM resulting from our ‘translation’ process. Along the way, we highlight the specific transformations needed to make the original ABM learnable. A more detailed explanation of the equations describing our learnable model is given in Materials and Methods. Supplementary Section S1 provides more details on the models and the translation process, and it reports the causal links between variables as a graphical model.

### Original ABM^13^

The ABM describes the housing market of a city composed of *L* locations or neighborhoods, each with a number of indistinguishable homes, inhabited by agents. Each agent belongs to an income class *k*, out of *K* income classes, each characterized by an income $Y_k$. At each time step, individual agents—represented as discrete units—choose a neighborhood to purchase a home if they act as buyers, or put their home on sale if they act as sellers. One fundamental insight encapsulated in this model is the formalization of the *attractiveness* of each neighborhood, which regulates how likely an agent is to bid for that location. The model assumes that the higher the income of residents, the more attractive a neighborhood is. In this original model, matching between individual buyers searching in a neighborhood and sellers in the same neighborhood is modeled as a *continuous double auction*. This process selects buyers and sellers sequentially at random, puts buyers in a queue ordered from highest to lowest bid price (and sellers from lowest to highest ask price), and, whenever a seller asks a price below the maximum bid price in the queue, matches the buyer with highest bid price to the seller with the lowest ask price. The social composition of the city evolves as a byproduct of these transactions, as high-income buyers may replace low-income sellers and lead to the gentrification of some neighborhoods. We report the pseudocode of this original model in Algorithm S1.

### Learnable ABM

In order to translate such ABM into a learnable model, we first rewrite it in terms of ‘counts’, i.e., instead of having variables for each individual agent, with a small loss of generality we consider the aggregated information about the number of identical agents of each income class in each location. This way, we obtain a model that revolves around the state variable $M_t$: at each time step, $M_t$ is a matrix of $L \times K$ entries, where $M_{t, x, k}$ represents the number of agents of income class *k* in location *x* at time *t*. Similarly, the number of agents of class *k* buying a house in location *x* is represented by $D^B_{t,x,k}$, giving a total of $D_{t, x}=\sum _k D^B_{t,x,k}$ transactions. $D_{t, x}$ is in turn determined as the short side of the market, i.e., the minimum between the number of sellers and the number of buyers in each case. While these two numbers in the original model were stochastic, in our learnable model we use a mean-field approximation, and replace the stochastic realizations with their expected value. The final key variable is $P_{t, x}$, which represents the average price of transactions that occur in location *x* at time *t*.

### Matching protocol

The matching protocol between buyers and sellers clearly exemplifies the type of transformations needed for our purpose. The continuous double auction of the original ABM is indeed hard to translate into a computationally tractable likelihood. First, we assume that we do not have detailed information on buyers and sellers for individual transactions, so estimating, e.g., the stochastic sequence in which buyers and sellers enter the queue is not feasible. Second, picking the buyer with highest proposed price is equivalent to an ${\text {argmax}}$ operation. Such operation is not differentiable, thus causing the whole likelihood to be not differentiable. Indeed, estimating its outcome would require enumerating all possible cases. To solve both issues, while preserving the properties of the model, we replace the continuous double auction by a multinomial distribution that gives higher probability of matching to buyers proposing higher prices. This rule is differentiable and can be estimated from observed prices: higher prices indicate that richer agents have settled in the neighborhood.

## Algorithms

Once we have translated the original ABM into its learnable counterpart, we design an algorithm that infers latent variables by maximizing the likelihood of these variables with respect to observed data and the model’s assumptions.

To start with, we need to determine which variables are observed and which are latent. To do so, we think of aggregate information about transactions as the only observable at our disposal. In particular, we assume to know, for each neighborhood and over time, the number of transactions $D_t$ and the average price $P_t$. Our key latent variable is instead $M_t$, the distribution of agents of each income class across neighborhoods. We believe this distinction to reflect a common real-world setting in economic models: one might have access to which actions are being performed, but not to the latent state of agents (e.g., where they live, and what is their income). As a matter of fact, in many countries it is relatively easy to obtain spatially granular data on transactions, but it is much harder to obtain such data on incomes^14^.

Note that $M_t$ can be computed deterministically given $M_{t-1}$ and $D^B_{t-1}$. Therefore, our problem reduces to finding an estimate for the latent stochastic variable $D^B_t$, over all time steps $t=1,\ldots ,T$, and for the starting condition $M_0$, given $P_t$ and $D_t$: all the other variables are in fact deterministic, and their value is fixed given the formers. This scenario corresponds to the graphical model shown in Materials & Methods.

However, the number of possible states of $D^B$ grows exponentially with the total number of time steps *T*: evaluating all possible paths of agents over all time steps would be unfeasible even for small values of *T*. Therefore, we approach our problem as an online task^15^, a common technique in machine learning in cases where processing the entire data set at once is unfeasible. We process the data per time step: at each *t*, the algorithm is presented with the newly observed values $D_t$ and $P_t$, and it updates its estimate of the latent variables $M_0$ and $D^B_t$, while considering all the values previously estimated as fixed. After the given time step *t* has been processed, the algorithm is applied on $t+1$, and so on until the last time step *T*. This process—examining each time step from $t=0$ to *T*—is iterated for a number of epochs: after the last time step *T* has been processed, the algorithm re-starts from the beginning, so that the first time steps are re-evaluated in light of successive ones.

To solve this likelihood optimization, we propose an expectation-maximization algorithm. Such an algorithm is able to obtain a maximum-likelihood estimate of the latent variables by optimizing the complete-data likelihood of the model. We outline its derivation in Materials & Methods “Algorithm derivation” section. It operates by repeating at each given time step *t* the following process. First, it evaluates the likelihood of each possible behavior of the agents—i.e, the possible outcomes of $D^B_t$; then, it uses back-propagation and online gradient descent to find the best $M_0$ under this likelihood. These two steps are alternated until convergence. This way, at each time step it recovers the most likely value for $D^B_t$, and it updates its estimate for $M_0$. All the other variables of the ABM are obtained deterministically from these ones.

In order for the algorithm described thus far to be scalable, we need to solve one last computational challenge: even in a single time step *t*, the space of possible outcomes of each $D^B_t$ is huge, since in principle one should consider the decisions of all individual buyers as independent. We solve this problem by considering that, while in an ABM simulation it is useful to model the behavior of individual agents, in fitting an ABM to data it is sufficient to evaluate the chances of *groups* of identical agents moving to one location or another: the behavior of a single agent is irrelevant with respect to the data we observe. Therefore, instead of considering all the possible outcomes of each $D^B_t$, we consider only those set apart by at least *s* agents, where *s* is a learning hyper-parameter.

## Experiments

In order to evaluate the efficacy of our approach, we perform two sets of experiments. First, we assess its fidelity, i.e., how well our method recovers latent variables. To do so, we generate a synthetic dataset from the original ABM as ground truth, and feed the observable part of such data to our likelihood-maximization algorithm. Second, we show that learning latent variables allows us to produce more accurate out-of-sample forecasts compared to existing heuristics.

### Recovering latent variables

We consider the time series of the price $P_{t,x}$ and of the number of transactions $D_{t,x}$ at each location *x* to be observable. We also assume that the macro-parameters that generated the data set (e.g., the total number of agents per location *N*, or the global income distribution) are known. Two other time series, namely that of inhabitants $M_{t,x,k}$ and buyers $D^B_{t,x,k}$, for all locations *x* and incomes *k*, are considered latent: they are hidden from the algorithm and used as a validation for what the algorithm learns.

We use the original ABM to generate 20 data traces with $L=5$ locations, $K=3$ income classes, and $T=20$ time steps that we use as ground truth. Each data trace differs from the others in the random initialization of $M_0$. We use the first 10 traces as training set to tune the hyperparameters of the algorithm (see Supplementary Figure S1). Then, on the remaining 10 traces that we hold out as test set, we evaluate the performance of the algorithm by computing the coefficient of determination $R^2$ between learned time series and ground truth ones. For each of the 4 variables listed above (*M*, $D^B$, *D*, *P*) and each of the 10 traces, we compute the $R^2$ by treating each variable as if it was a one-dimensional vector, thus comparing the ground truth with its estimate element-wise. Note that our learning algorithm uses the data from the learnable ABM to specify the likelihood, so there is some misspecification compared to the original ABM used to generate the ground truth. For completeness, we also repeat the same evaluation by using the learnable ABM to generate ground-truth data traces, thereby removing misspecification. We view this latter test as a sanity check for the algorithm.

**Figure 2:**
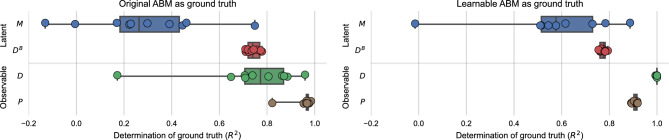
Quality of estimation in synthetic experiments with traces generated by the original (left) and learnable (right) ABM. For each variable, we report the coefficient of determination $R^2$ between the original values and the estimates for each trace. We represent each trace as a dot, with whisker plot as a summary for each variable. Whiskers extend from the minimum to the maximum value, while boxes range from the 25th to the 75th percentile.

Figure 2 shows the results for the 10 traces in the test set. As expected, our ability to reconstruct latent variables is higher for traces generated with the learnable ABM, as there is no misspecification. Perhaps more interestingly, our algorithm reconstructs the time series of buyers $D^B_{t,x,k}$ with higher fidelity than the time series of inhabitants $M_{t,x,k}$ (mean determination coefficient $R^2=0.74$ vs. $R^2=0.28$ with traces from the original ABM). Even though $M_{t,x,k}$ proves to be harder to reconstruct, we still obtain an informative estimate, that further improves when misspecification is removed ($R^2=0.57$). We conjecture that this difference in results may be due to the fact that $D^B_{t,x,k}$ is a “flow” variable that does not depend explicitly on previous time steps, while $M_{t,x,k}$ is a “stock” variable that depends on the whole history, so errors in estimating $M_{t=0}$ accumulate at following time steps. These results also hint at an identification problem in the original ABM, which we elaborate on further at the end of this section. Regarding the observable variables, our algorithm fits the prices $P_{t,x}$ almost perfectly ($R^2=0.95$), and the number of transactions $D_{t,x}$ very well ($R^2=0.74$). Without misspecification, the fit for the number of transactions is perfect ($R^2=1.0$). While such a good fit for observable variables is expected, since our inference method works by minimizing the distance from observable variables, this result indicates that there is no major misspecification introduced by using the learnable ABM to infer latent variables from traces of the original ABM. In other words, our translation does not alter the nature of the original model.

**Figure 3:**
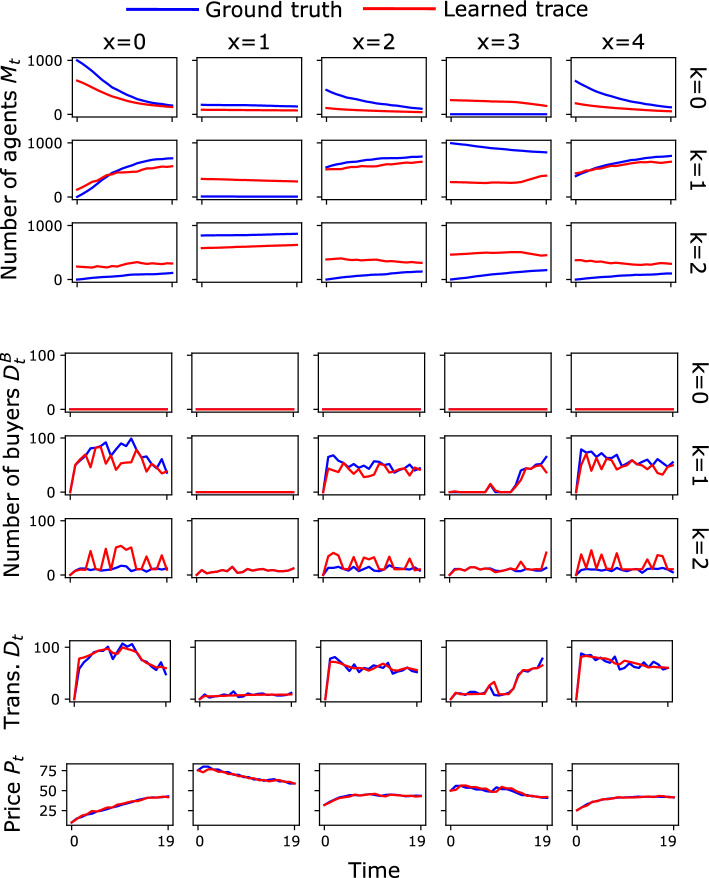
Estimates for $M_{t,x,k}$, $D^B_{t,x,k}$, $P_{t,x}$, $D_{t,x}$ compared to the traces generated with the original ABM, in a single experiment, chosen as the median experiment in terms of estimation quality.

Figure 3 zooms in on a representative trace generated by the original ABM. For fairness, we choose this experiment as the median one in terms of performance (i.e., correlation between the ground truth and estimated values of *M*). These time series confirm the intuition from Fig. 2: our approach is able to reconstruct $P_t$ and $D_t$ extremely well and it is also quite precise at reconstructing $D^B_t$. Our estimate of $M_t$ is also very accurate in most cases, but imprecise estimates of the initial conditions $M_0$ lead in a few cases (for instance, in location $x=3$) to an inaccurate reconstruction. In a few cases, in fact, the algorithm finds a local minimum that does not correspond to the ground truth.

One of the possible reasons behind this behavior is the presence of an *identification problem*. We show in fact that, in some cases, the likelihood of the observed data is the same for different possible values of the latent variable $M_0$. While these possible values include the ground truth (or, in case of misspecification, values extremely close to it) the model does not have enough information to distinguish it from the other possible optimal values of $M_0$. This phenomenon is intrinsic to the ABM under study, once we identify *P* and *D* as observable and $M_0$ as latent. We provide a concrete example in Supplementary Section S2.3. Figure S5 shows a representation of the likelihood able to efficiently visualize such issues. Our approach allows in fact to formally define and thus diagnose such issues. Of course, one could also do this by sampling from the parameter space and computing summary statistics, as with Approximate Bayesian Computation (ABC) calibration methods (see, e.g., Ref.^16^). Our approach, which features a closed-form of the likelihood, has three advantages over these methods: (*i*) avoidance of sampling error; (*ii*) higher efficiency, as we do not need to repeatedly execute the model; and (*iii*) the possibility to look for local minima using gradient-based methods.

### Out-of-sample forecasting

Except for a few recent attempts^5,17,18^, so far the use of ABMs for forecasting has been limited. A key problem is that ABM state variables are mostly latent, as it is often hard to observe information that describes individual agents. To the extent that the aggregate dynamics depends on the agent states, a wrong initialization of the latent state variables is likely to lead to a very inaccurate forecast. In this section, we explicitly test whether this is true for our model by using synthetic experiments.

To shift our focus away from misspecification errors, we use the learnable ABM to generate the ground truth. We extend each of the 10 test traces for 5 additional time steps, so that the total length of each simulation becomes $T'=25$. For each trace, starting from the true state, we repeat the simulations of the additional 5 time steps 100 times. We take the ensemble average of the time series as the ground truth, thus obtaining a single time series that represents the expected evolution of the system. This way, the ground truth is not influenced by stochastic noise due to sampling.

Our protocol for the various forecasting methods is the following. We initialize the learnable ABM at $t=20$ with a given estimate of the latent state variables $M_{T=20}$, and let it produce the time series $P_t$ and $D_t$ for the out-of-sample time steps $t \in [21,25]$. As in the ground truth, we repeat the simulations 100 times and take the average, thus removing stochastic effects. We compare five approaches for the estimate of the latent variable $M_T$:

1. *Random*: we draw $M_T$ from a Dirichlet distribution whose parameters are consistent with the share of buyers $\Gamma _k$. A random initialization of latent variables is very common in ABMs, for instance in epidemiological ABMs it is common to choose infected seeds at random^19^.
2. *Proportional*: we draw $M_T$ in a way that locations with price higher than the mean price over the city have a higher share of high-income inhabitants. The strength of this relation is governed by a hyperparameter that we calibrate in-sample on the same 10 traces that we use to select the hyperparameters of the learning algorithm.
3. *Time series*: we run 1000 simulations starting from different values of $M_0$ and select the $M_T$ corresponding to the simulation with the lowest RMSE with respect to the observable time series $P_t$ and $D_t$ in sample, i.e., for $t \in [1,20]$. This is, for instance, the method used by Geanakoplos et al.^20^.
4. *Constant predictor*: we also compare the forecasts obtained by simulating $P_t$ and $D_t$ with different estimates of $M_T$ to a constant predictor, i.e., $P_t=P_T$ and $D_t=D_T$, $t=21,\ldots , 25$.
5. *Learnable*: we infer $M_T$ with our algorithm by using the estimates obtained as specified in the previous section.

**Figure 4:**
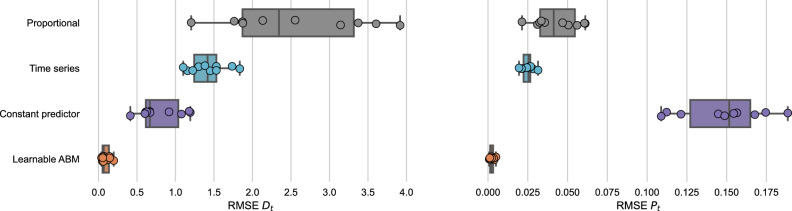
Out-of-sample forecasting error for our method compared to alternative benchmarks for the number of transactions $D_t$ (left) and prices $P_t$ (right). We show the forecasting error as the RMSE of each time series. We consider the same 10 traces as in the experiments above, and show results for each of the 10 traces as a dot and a whisker plot as a summary. Whiskers extend from the minimum to the maximum value, while boxes range from the 25th to the 75th percentile.

To evaluate the quality of the forecasts obtained by these approaches, we compute the Root Mean Squared Error (RMSE) for the observable time series $P_t$ and $D_t$, summing the errors from time step $t=21$ up to $t=T'=25$. Figure 4 shows the results. We do not show the values for the *Random* approach as it is well above that of the other approaches (RMSE $P_t \approx 0.3$, RMSE $D_t \approx 12$). The *Constant predictor* approach performs better than the *Proportional* and *Time series* approaches for $D_t$, but not for $P_t$. Most importantly, the *Learnable* approach substantially improves over the *Proportional*, *Time series*, and *Constant predictor* approaches for both $P_t$ and $D_t$. This is a strong improvement that shows the potential of our method in enhancing the forecasting capabilities of ABMs.

However, we believe that the value of our approach goes beyond this aspect. In fact, alternative approaches are heuristics, that do not yield much insights about inference. By contrast, our approach is more principled: it frames the problem of estimating unobservable variables of an ABM into probabilistic inference. This methodology opens new research directions to further improve our results. For instance, designing learnable ABMs from the start, for which there would be no misspecification error. Even more importantly, it makes it possible to formally reason about the likelihood of an ABM—for instance, to spot potential identification problems.

## Discussion

Our proposal aims to integrate the benefits of agent-based modeling with the capability to fit to data. We assert that certain systems necessitate an ABM for an accurate representation as other methods may lack the ability to incorporate established micro-level causal mechanisms, such as behavioral patterns, as priors. Our methodology strives to develop a *learnable* ABM that not only represents these rules, but also has a well-defined likelihood and can be fitted to data. By doing so, we can create a more credible and accurate representation of the system’s behavior, which is essential for policymakers and other decision-makers.

From the specific translation of the housing market ABM considered in this paper, we can identify some general design principles, that we believe will be useful in making other ABMs learnable, and eventually lead to a streamlined process. First of all, it was necessary to tune the level of stochasticity of the model by considering which variables are observed and which are latent. In most graphical models, latent variables are stochastic random variables that are related to the observable ones—indeed, if they were deterministic, they could be computed exactly^21^. All stochastic variables that are not observed must be estimated, thus increasing both the computational complexity of the process and the uncertainty of the model. However, in our translation, we have room to decide which variables are deterministic and which are stochastic. To make the model truly learnable, we need to balance observable and latent variables so that for every latent variable we have some observable that intuitively makes it possible to estimate it. We can encapsulate this first design principle as follows.

### Principle 1

**Stochasticity parsimony** In a learnable ABM, the amount of stochasticity should be commensurate to data availability.

Second, we needed to carefully consider which variables and functional forms should be discrete. ABMs often consist of discrete units, and it is common for agents to choose between different discrete possibilities. However, discreteness makes likelihood optimization problematic. Indeed, whenever we deal with discrete variables, the likelihood must consider all possible combinations of values for discrete stochastic variables, which greatly increases the computational burden of the approach. Moreover, in some cases the likelihood may be flat over some region of the latent space, thus hindering the progress of optimization algorithms such as gradient descent. Given these considerations, it is important to limit the use of discrete variables to the ones that are critical to the behavior of the model.

### Principle 2

**Differentiability preference** A learnable ABM should prefer continuously differentiable functions over discrete choices when they do not alter the behavior of the model.

Following these principles, it should be possible to transform any ABM into a learnable one, given enough data. While the translation in this paper was still hand-curated, it is a first step towards its proper formalization, and thus automatization.

Nevertheless, different alternative methods have been suggested in the literature to obtain similar results. Making the state of the system compatible with real-world observations has traditionally been the goal of data assimilation techniques, such as the various versions of the Kalman filter or the particle filter. Originally developed in meteorology and geosciences^22^, data assimilation techniques have recently been employed in ABM research^4,9–11^. These works treat the ABM as a black box, adjusting ABM state variables so that forecasts come closer to observations. The main advantage of data assimilation techniques over our approach is that they do not require building a new model (the learnable ABM). At the same time, our approach offers several advantages.

(i) It deals with the estimation of discrete variables in a natural and principled way. Standard data assimilation methods only allow to tune continuous variables^4,9^, and recent attempts to deal with discrete variables^10,11^ tend to be heuristic and problem-specific.
(ii) Its closed-form likelihood can be maximized with computationally efficient gradient-based methods, by leveraging deep learning frameworks/architectures.
(iii) Such closed-form likelihood is also an essential tool to analyze identification problems, thus offering explanations about the estimated variables.
(iv) While Kalman filters require Gaussian or quasi-Gaussian noise, and linear or weakly non-linear functional forms, our approach can easily integrate most types of stochastic element and non-linearities.

Considering these advantages, we believe that likelihood-based estimation of ABM micro-states is a promising direction to obtain more principled approaches to data-driven ABMs.

## Conclusions

In this work, we have shown how to translate a complex agent-based model into a probabilistic graphical model to obtain a *learnable* ABM that can be fit to data. By employing techniques such as maximum likelihood estimation, we can estimate the latent micro-state variables of the agents in a way that is consistent with both the model and observed data. To accomplish this, we developed an expectation-maximization algorithm that estimates the latent variables given the observed ones. We have demonstrated that this process is effective in recovering unobserved variables that are consistent with both the learnable and original models, across a range of scenarios. This process enables us to incorporate learned variables into the ABM, thus resulting in a simulation of the micro-states that accurately reflects the provided data. The resulting methodology provides a powerful tool for using ABMs in forecasting and policy design.

Building a fine-grained link between an ABM and observed data opens the way for different exciting opportunities. As we have shown in this work, it allows in the first place for better usage of ABMs as tools for prediction. Initializing agents’ micro-states in a way that is coherent with observed data ensures that their future trajectory can be regarded as the best compromise between the theoretical assumptions of the model and the available observations. Consequently, the quality of predictions serves as a direct validation (or falsification) of the causal model embodied in the ABM. Besides these immediate advantages, the approach provides opportunities for more advanced applications. For instance, defining the likelihood of the model w.r.t. the observations enables model selection by using available data. In other words, it allows using ABMs to formulate hypotheses and test them against real data as has been demonstrated in simpler cases^15^. The extension of this technique to more complex ABMs requires further analysis and opens avenues for novel research directions. Moreover, the translation of an ABM into a probabilistic model compels the modeler to reveal their assumptions and consider the inferential problem. Consequently, it highlights potential identification issues. For instance, when different models or parametrizations of the same model produce the same observable state, how can one choose the correct one in practice? Such a problem, frequently ignored in ABM research, will be crucial to address in future applications of ABMs to real-world data.

Our approach for creating learnable ABMs builds upon the framework of probabilistic graphical models^23^, which offers exciting opportunities for interdisciplinary collaboration but also presents new theoretical challenges. Due to the complexity of ABMs, many commonly used methods such as Markov Chain Monte Carlo (MCMC)^23^ become computationally infeasible. ABMs model emergent behavior through the combination of many simple rules, often involving long chains of dependencies among variables, with highly non-linear behavior. Hence, many theoretical properties necessary for the convergence of MCMC, such as the uniqueness of the posterior distribution^24^, may be absent. Furthermore, the posterior distribution is often very complex and high-dimensional, and thus challenging to learn via sampling techniques. Thus, we choose to maximize the likelihood by using gradient descent and automatic differentiation^25^. Interestingly, the long sequences of deterministic transformations in ABMs make our optimization task similar to deep learning. However, while the transformations in deep learning are purely data-driven, aiming only to maximize prediction accuracy, our methodology still emphasizes causal mechanisms: each transformation represents an aspect of the theory being modeled.

## Materials and methods

### Model description

Here, we give a minimal description of the learnable model. In Supplementary Section S1.2 we provide a longer description as well as a detailed interpretation of each modeling assumption.

**Figure 5:**
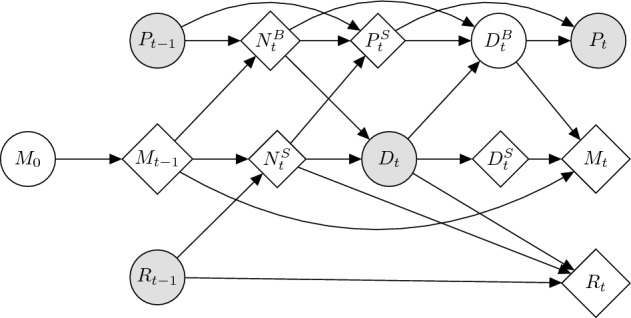
Graphical model diagram of the learnable ABM for a time step *t*. See Materials & Methods “Model description” section for notation. Diamonds indicate deterministic variables, white circles indicate latent stochastic variables, grey circles indicate observed stochastic variables.

The model represents the housing market of a city with *L* locations or neighborhoods denoted by $x=1,\ldots ,L$, each with *N* indistinguishable homes, inhabited by agents that are only distinguished by their income class $k=1,\ldots ,K$. The vector of state variables $Z_t$ is composed by the variables $\left\{ M_{t,x,k}\right\} ,\left\{ P_{t,x}\right\} ,\left\{ R_{t,x}\right\}$, where $M_{t,x,k}$ is the number of agents of income *k* living in location *x* at time *t*; $P_{t,x}$ is the average price of location *x* at time *t*; and $R_{t,x}$ is the inventory of unsold homes at location *x* at time *t*. These state variables are updated according to deterministic and stochastic equations that represent the demand and supply sides of the housing market, and the matching between potential buyers and sellers. The causal links between these variables are summarized in Fig. 5. All the equations of the model, Equations (M1) to (M13), are listed in Table 1.

**Table 1:**
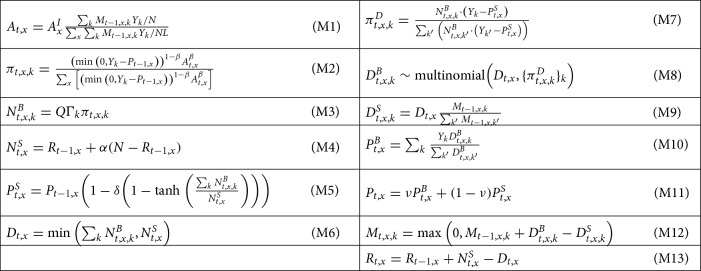
Equations defining our agent-based model.

Equations (M1) to (M3) characterize the number of buyers from each income class that try to buy a house at each location at time *t*. Buyers prefer to live in locations with higher attractiveness $A_{t,x}$, which depends on a constant local intrinsic attractiveness $A_x^I$ and on the time-varying average income at that location, captured through $Y_k$—the income of agents in income class *k* (M1). However, locations with high attractiveness may also be more expensive, so the probability $\pi _{t,x,k}$ for a buyer of income class *k* to choose location *x* also depends on the difference between their possibility to pay—here exemplified by income $Y_k$—and price $P_{t,x}$ (M2). Finally, the number of potential buyers of income class *k* for location *x* at time *t*, $N^B_{t,x,k}$, is given by simply assuming that, for each income class *k*, a fraction $\Gamma _k$ of the total buyers *Q* distribute themselves among all locations following probabilities $\pi _{t,x,k}$ (M3).

Next, Equations (M4) to (M5) characterize the supply side of the market. The number of sellers $N_{t,x}^S$ is given by the inventory of houses on sale at the previous time, $R_{t-1,x}$, plus a fraction $\alpha$ of the houses that were not on sale (M4). Moreover, the minimum price that the sellers at location *x* are willing to accept, $P^S_{t,x}$, is a smooth function of the ratio between the number of buyers and sellers at *x*: when there are more buyers than sellers, sellers refuse to sell at a price below $P_{t-1,x}$; conversely, when there are more sellers, they are willing to sell at a discount, up to a price that is $1-\delta$ of $P_{t-1,x}$ (M5).

The demand and supply sides of the market are matched in Equations (M6) to (M11). The number of deals or transactions $D_{t,x}$ is the short side of the market, i.e., the minimum between the number of buyers and sellers (M6). When there are more buyers than sellers, only some buyers are able to secure a deal. The probability that agents from income class *k* secure a deal at *x* is represented by $\pi _{t,x,k}^D$, which is proportional to the number of buyers from class *k* and to their income (M7). The number of buyers of each income class who secure a deal, $D_{t,x,k}^B$, is given by the $D_{t,x}$ realizations of a multinomial with parameter $\pi _{t,x,k}^D$ (M8). In this way, the outcome of this random variable has to be consistent with $D_{t}$: the total number of buyers in each location *x* is fixed to $D_{t,x}$; for each location, the buyers are distributed among income classes according to $\pi _{t,x}^D$. The number of sellers from class *k* who manage to sell at location *x* ($D_{t,x,k}^S$) is instead simply proportional to the fraction of *k*-agents living in location *x* (M9). With Equations (M6) to (M9) having determined the income classes of buyers and sellers, Equations (M10) and (M11) specify the average (observed) price of transactions $P_{t,x}$. The model assumes that this is a weighted mean (M11) of the maximum price that the average buyer is willing to pay, $P^B_{t,x}$ (M10), and of the minimum price that sellers are willing to accept, $P^S_{t,x}$ (M5).

As a last step, we update the remaining state variables in Equations (M12) and (M13), simply by tracking the number of buyers and sellers in each class and location.

### Algorithm derivation

Here, we provide a more detailed description of our algorithm. Following our online assumption, its goal is to estimate latent variables at time *t* by looking at observables at the same time step, and treating all the previously estimated variables as fixed. Specifically, $D^B_0, \dots , D^B_{t-1}$, i.e. the buyers who previously relocated, and the corresponding sellers $D^S_0, \dots , D^S_{t-1}$, are fixed. Therefore, the algorithm observes $P_t$ and $D_t$, and then it provides a new estimate of $D^B_t$, and update the estimate of $M_0$. In fact, since previous $D^B$ and $D^S$ are fixed, $M_t$ is a deterministic function of the latent variable $M_0$

$$\begin{aligned} M_{t+1} = M_t + D^B_{t} - D^S_{t} = M_0 + \sum _{\tau = 0}^{t} \left( D^B_{\tau } - D^S_{\tau } \right) \end{aligned}$$

allowing us to treat $M_0$, and not $M_t$, as our latent variable.

Since our observed variables are in principle also a deterministic result of the others, we model their observed value as a noisy proxy of the value determined by the agent-based model rules. Specifically, for prices we assume that we observe $\tilde{P}_t$, given by $\tilde{P}_t=P_t + \epsilon _P$, where the error $\epsilon _P$ is normally distributed and $P_t$ is the deterministic estimate of prices as computed by the model (see (M11)). Similarly, the number of observed deals $\tilde{D}_t$ will follow $\tilde{D}_t = D_t + \epsilon _D$.

Now, computing the likelihood of the observed prices $\tilde{P}_t$ requires knowledge of the latent variable $D^B_t$, that is, the distribution of buyers among classes and locations, which is a discrete outcome of a stochastic process dependent on our main latent variable $M_0$. Therefore we resort to the (Generalized) Expectation Maximization algorithm. In this way, we alternate between evaluating the expectation of $D^B_t$ and updating our estimate of $M_0$ under the current estimate of expectation. The latter can be performed with online gradient descent, since—once we fixed the probability of each possible outcome of $D^B_t$—what remains of the likelihood is a continuous and differentiable function of $M_0$.

First, observe that $\tilde{P}_t$ and $\tilde{D}_t$ are independent given $M_0$. In fact, $D_t$ is fixed given $M_0$; the distribution of $D^B$ is also fixed, since it is determined from $D_t$ and $\pi ^D$; and the error $\epsilon _D$ is drawn independently from the extraction of $D^B$ from such distribution and from $\epsilon _P$. Therefore we can factorize the complete-data likelihood w.r.t. observed data $\mathbb {D} = \left\{ \tilde{P}_t, \tilde{D}_t \right\}$ as:

2 $$\begin{aligned} \mathbb {P}\left( \mathbb {D} | M_0\right) = \mathbb {P}\left( \tilde{P}_t | M_0\right) \cdot \mathbb {P}\left( \tilde{D}_t | M_0\right) . \end{aligned}$$

Since computing $\mathbb {P}\left( \tilde{P}_t | M_0\right) = \sum _{D_b} \mathbb {P}\left( D_b | M_0\right) \mathbb {P}\left( \tilde{P}_t | D_b, M_0\right)$ without the knowledge of the latent variable $D^B_t$ would be unfeasible, we resort to the Generalized Expectation Maximization algorithm, alternating these two steps until convergence:

1. First, we evaluate the expectation of $D^B_t$ given the rest of the variables. Given the set $\Omega$ of all possible values of $D^B_t$, for each $D^B_t \in \Omega$ we evaluate
  $$\begin{aligned} q\left( D^B_t\right) := \mathbb {P}\left( D^B_t | M^*_0\right) \end{aligned}$$
  where $M^*_0$ is the current estimate of $M_0$. This probability is computed from (M8).
2. Then, we update the estimate of $M_0$ in order to increase the likelihood from Equation (2), by increasing the auxiliary function
  3 $$\begin{aligned} \mathbb {Q}\left( M^*_0\right) := \sum _{D^B_t \in \Omega } q(D^B_t) \, \log \mathbb {P}\left( \tilde{P}_t, \tilde{D}_t, | D^B_t, M^*_0 \right) \end{aligned}$$

Noting that $\tilde{D}_t$ does not depend on $D^B_t$ (see Fig. 5), the last probability can be decomposed as

4 $$\begin{aligned} \log \mathbb {P}\left( \tilde{P}_t, \tilde{D}_t | D^B_t,M^*_0 \right) = \log \mathbb {P}\left( \tilde{P}_t | M^*_0, D^B_t\right) + \log \mathbb {P}\left( \tilde{D}_t | M^*_0\right) . \end{aligned}$$

These two elements are given by the gaussian distribution of the errors $\epsilon _P, \epsilon _D \sim \mathcal {N}(0, \sigma )$ ($\sigma$ being a hyper-parameter), between $P_t$ and $\tilde{P}_t$, and $D_t$ and $\tilde{D}_t$ respectively. Note that *P* and *D* are a deterministic function of the latent variable $M_0$ we are optimizing, through the chain of deterministic equations of the ABM (Table 1). The only free variable is in fact $M_0$, since the previous variables from time steps $t' < t$ are assumed to be fixed, and the value of $D^B$ is known for the assumption of EM. Therefore, since all these deterministic functions are continuous and differentiable in the general case, it is easy to update $M^*_0$ ascending the gradient $\nabla _{M^*_0}\mathbb {Q}\left( M^*_0\right)$. The complexity of computing this gradient is left to differentiable programming frameworks.

Nevertheless, this approach presents a problem: the set $\Omega$ of possible values for $D^B_t$, the matrix of numbers of actual buyers for each class and each location, is potentially huge (precisely, $\genfrac(){0.0pt}1{n+k-1}{n}$ with *n* buyers and *k* classes). We solve this problem with two considerations: first, we will show that $\tilde{P}_{t, x} \perp \!\!\! \perp \tilde{P}_{t, y} | D_{t}, \pi ^D_{t}$; second, we are not interested in all the possible values for the number of actual buyers: the behavior of a single agent is irrelevant with respect to the data we observe. Let us analyze these two key points.

The first consideration stems from the the independence of outcome in different neighborhoods: $D^B_{t, x} \perp \!\!\! \perp D^B_{t, y} | D_{t}, \pi ^D_{t}$. This fact follows naturally from (M8), since all locations are independently drawn. As a consequence, also the probability of observed prices $\tilde{P}_{t, x}$ and $\tilde{P}_{t, y}$ are independent from each other for any two locations $x \ne y$, since (M10) and (M11) do not have any inter-location effect, and the observation noise $\epsilon _P$ is also independent across locations. Therefore $\tilde{P}_{t, x} \perp \!\!\! \perp \tilde{P}_{t, y} | D_{t}, \pi ^D_{t}$ and we can write:

5 $$\begin{aligned} \mathbb {P}\left( \tilde{P_{t}}|M_0, D^B_{t}\right) = \prod _{x} \mathbb {P}\left( \tilde{P}_{t, x} | M_0, D^B_{t, x}\right) \end{aligned}$$

Thus, we can factorize Eq. (3) in a more practical way. Let us call $\Omega _x$ the set of all possible values for $D^B_{t, x}$, given a location *x*. Then, our algorithm becomes an iteration of the following two steps until convergence.

1. Evaluate $$\forall x \in \{1, \dots , L \}$$ and $$\forall D^B_{t, x} \in \Omega _x$$:
  6 $$\begin{aligned} q\left( D^B_{t, x}\right) := \mathbb {P}\left( D^B_{t, x} | M^*_0\right) . \end{aligned}$$
  Note that any two values in $\Omega _x$ are mutually exclusive, so $\sum _{D^B_{t, x} \in \Omega _x} q(D^B_{t, x}) = 1$ holds for all *x*.
2. Update $M^*_0$ by ascending the gradient $\nabla _{M^*_0}\mathbb {Q}(M^*_0)$ of
  $$\begin{aligned} \mathbb {Q}(M^*_0): = \sum _{x} \sum _{D^B_{t, x} \in \Omega _x} q\left( D^B_{t, x}\right) \, \log \mathbb {P}\left( \tilde{P}_{t, x}, \tilde{D}_t | M^*_0, D^B_{t,x} \right) \end{aligned}$$
  $$\begin{aligned} = \log \mathbb {P}\left( \tilde{D}_t | M^*_0\right) + \sum _{x} \sum _{D^B_{t, x} \in \Omega _x} q\left( D^B_{t, x}\right) \, \log \mathbb {P}\left( \tilde{P}_t | M^*_0, D^B_{t,x}\right) \end{aligned}$$
  because of Eqs. (4) and (5).

To define the set $\Omega _x$ we take advantage of the second key point: two different values of $D^B_{t, x}$ might be indistinguishable in practice given our data, if they differ only by a few agents. Thus, instead of considering all the possible partitions of the integer $D_{x,t}$ in *K* positive integers, we only consider their quotient for a given constant *s* (i.e., $\left\lfloor D_{x,t} / s \right\rfloor$): this can be thought of as the possible outcomes obtained by moving *groups* of *s* agents at a time. Any difference below *s* is considered negligible. In practice, we set *s* as a consequence of the available memory. Given a maximum size for $|\Omega |$, we set a threshold for each $|\Omega _x|$ proportional to its original size $\genfrac(){0.0pt}1{n+k-1}{n}$. Here, we keep in consideration the effective number of classes $k \le K$ that can afford a location, since both $P_{t-1}$ and *Y* are assumed to be known at time *t*. After setting this threshold, we find the minimum *s* s.t. $|\Omega _x|$ respects such threshold.

## Supplementary Information

Supplementary Information.

## Data Availability

The datasets generated and analysed during the current study are available in the repository: https://github.com/corradomonti/data-driven-econ-abm.
