# Supplementary material for: On learning agent-based models from data
Source: Sci Rep. 2023 Jun 7;13:9268. doi: 10.1038/s41598-023-35536-3 (PMC10247821; doi:10.1038/s41598-023-35536-3)
Supplement: Supplementary file 1 — Supplementary Information. [file 41598_2023_35536_MOESM1_ESM.pdf]

Supplementary Information

S1 Original vs. learnable ABM

This section provides more details about the original ABM (Section S1.1), the learnable ABM (Section S1.2), and the comparison between the two (Section S1.3).

S1.1 The original ABM

Pangallo et al. [1] introduce an ABM that describes the housing market of a city. It is beyond the scope of this section to fully repeat the description of the model and to justify each assumption, so we give a brief overview and report the pseudocode of the model (see Algorithm S1 and Algorithm S2).

The city has $N \cdot L$ inhabitants (see Table S1 for a summary of the notation used), with Q buyers coming to the city every time step to purchase a home, divided between γ_k buyers belonging to income class k, $k = 1, \ldots K$, such that $Q = \sum_k \gamma_k$. Each inhabitant i is characterized by four state variables that can change over time t: state $s_{t,i}$ (buyer, housed, seller), reservation price $P^R_{t,i}$, location $x_{t,i}$, and a categorical income $Y_{t,i}$, that belongs to one of the K income classes. The city is composed of L neighborhoods or locations x that are distinguished by their intrinsic attractiveness A^I_x, social attractiveness $A^S_{t,x}$ (which depends on which agent inhabit the neighborhood), and market price $P_{t,x}$.

The model is initialized following some protocol to locate agents with different income in the city (e.g., uniformly at random, or following some predefined spatial distribution). After that, at each time step t some buyers come to the city to purchase a home, some housed agents decide to leave and sell their home, and buyers and sellers at each location are matched via a continuous double auction.

Algorithm S1 details the operations that occur at each time step. First, the model updates some location-specific variables which reflect the change in social composition that occurred in the previous time step (lines 1 to 9). These include updating the average income and social attractiveness at each location and then computing the utility for agents of a given income class at a given location. Next, buyers choose a location where they try to purchase a home (lines 10 to 22). After that, housed agents may put their home on sale with probability α and set a reservation price by applying a markup μ to the market price of the location where they live (lines 23 to 29). Agents that decided to sell their home in previous time steps but were unsuccessful reduce their price by a factor λ every τ time steps (lines 30 to 34). Finally, buyers and sellers are matched at each location via a continuous double auction, and successful buyers replace successful sellers (lines 35 to 37).

Algorithm S2 details the continuous double auction process. At each location, there is a set of buyers $\mathcal{B}_{t,x}$ and a set of sellers $\mathcal{S}_{t,x}$ (lines 1 to 2). If there are in fact either no buyers, or no sellers, or all reservation prices of the buyers are lower than the reservation prices of the sellers, no transaction takes place, and the market price does not get updated (lines 3 to 4). If instead at least one transaction can occur (lines 5 to 23), the following process takes place. First, one creates auxiliary lists of buyers and sellers (also known as *logs*), $\mathcal{O}^B_{t,x}$ and $\mathcal{O}^S_{t,x}$ respectively, and fills them as agents are drawn uniformly at random from the common pool of buyers and sellers. Every time that a buyer with a higher reservation price than a seller enters $\mathcal{O}^B_{t,x}$ (or a seller with a lower reservation price than a buyer enters $\mathcal{O}^S_{t,x}$), the buyer with highest reservation price is matched with the seller with lowest reservation price. The price of the transaction is the weighted mean of the respective reservation prices, with weight given by a parameter ν capturing bargaining power, and this individual transaction price is added to the list $\mathcal{P}_{t,x}$. Finally, the seller leaves the city and the buyer settles where the buyer was. The market price is computed as the mean of the transaction prices.

Table S1: Notation used by Pangallo et al. [1]. The first block is indexes, the second block global parameters, the third block agent attributes, the fourth block location attributes.

Type	Variable	Meaning
Indexes	t	Time
	i, j, h, ι	Agent
	k, κ	Income class
	x	Location
Global parameters	N	Number of agents/housing units at any location
	L	Number of locations
	K	Number of income classes
	$A^{\text{I, max}}$	Maximum intrinsic attractiveness
	R	Radius intrinsic attractiveness
	P_0	Initial price that is the same at all locations
	β	Preference for housing goods (vs. non-housing goods)
	α	Probability to put house on sale
	μ	Markup
	λ	Reduction in reservation price if sale unsuccessful
	τ	Time steps of unsuccessful sale needed to reduce reservation price
	ν	Bargaining parameter
	Y_k	Income of class k
	γ_k	Number of incoming agents of class k at any time step
Agent	$s_{t,i}$	State of agent i. $s_{t,i} = 0$: Buyer. $s_{t,i} = 1$: Housed. $s_{t,i} = 2$: Seller
	$x_{t,i}$	Location where agent i searches if $s_{t,i} = 0$, otherwise location where it lives if $s_{t,i} = 1, 2$
	$P^R_{t,i}$	Buyer reservation price of agent i if $s_{t,i} = 0$, seller reservation price if $s_{t,i} = 2$
	$Y_{t,i}$	Income of agent i
	t^S_i	Time when agent i becomes a seller
Location	d_x	Distance of location x to the center
	$\overline{Y}_x$	Mean income at location x
	$P_{t,x}$	Price of location x at time t
	$A_{t,x}$	Attractiveness of location x at time t
	A^I_x	Intrinsic attractiveness of location x
	$A^S_{t,x}$	Social attractiveness of location x at time t
	$U_{t,x,k}$	Utility for k-buyers at location x at time t

Algorithm S1 Original model run at time step t

Input: Global parameters $N, L, K, \beta, \alpha, \mu, \lambda, \tau, \nu, \{Y_k, \gamma_k\}_{k=1}^{K}$, $\{A_x^I\}_{x=1}^{L}$
Agent variables $\{s_{t-1,i}, P_{t-1,i}^R, x_{t-1,i}, Y_{t-1,i}\}_{i=1}^{N^{\mathrm{TOT}}}$, location variables $\{P_{t-1,x}\}$
Output: Agent variables $\{s_{t,i}, P_{t,i}^R, x_{t,i}, Y_{t,i}\}_{i=1}^{N^{\mathrm{TOT}}}$, location variables $\{P_{t,x}\}$

$\overline{Y}_t = \sum_{i=1}^{N^{\mathrm{TOT}}} Y_{t,i}/N^{\mathrm{TOT}}$ ▷ Compute average income over the city
for $x = 1, \dots, L$ **do** ▷ Update attractiveness and utility
  $\overline{Y}_{t,x} = \sum_{i \text{ s.t. } x_{t,i}=x} Y_{t,i}/N$ ▷ Compute average income at location x
  $A_{t,x}^S = \overline{Y}_{t,x}/\overline{Y}_t$
  $A_{t,x} = A_x^I \cdot A_{t,x}^S$
  for $k = 1, \dots K$ **do**
    $U_{t,x,k} = \begin{cases} (Y_k - P_{t-1,x})^{1-\beta} (A_{t,x})^{\beta} & \text{if } Y_k - P_{t-1,x} > 0 \\ 0 & \text{if } Y_k - P_{t-1,x} \leq 0 \end{cases}$
  end for
end for
for $x = 1, \dots, L$ **do**
  for $k = 1, \dots K$ **do**
    $\pi_{t,x,k} = \frac{U_{t,x,k}}{\sum_x' U_{x',k,t}}$ ▷ Probability that k-buyers choose location x
  end for
end for
for $k = 1, \dots, K$ **do** ▷ Create buyers and let them choose a location
  for $i = N^{\mathrm{TOT}} + \sum_{\kappa=1}^{k-1} \gamma_\kappa + 1, \dots, N^{\mathrm{TOT}} + \sum_{\kappa=1}^{k-1} \gamma_\kappa + \gamma_k$ **do** ▷ Do not duplicate id i
    $s_{t,i} = 0$ ▷ Buyer
    $Y_{t,i} = Y_k$
    $P_{t,i}^R = Y_k$ ▷ Buyer reservation price = income
    $x_{t,i} \sim \text{Categorical}\left(\pi_{t,1,k}, \dots, \pi_{t,L,k}\right)$
  end for
end for
for i s.t. $s_{t-1,i} = 1$ **do** ▷ Housed agents put their home on sale with probability α
  if Bernoulli$(\alpha) = 1$ **then**
    $s_{t,i} = 2$ ▷ Seller
    $t_i^S = t$
    $P_{t,i}^R = (1+\mu) P_{x_{t,i},t-1}$ ▷ Reservation price is a markup μ over previous market price
  end if
end for
for i s.t. $s_{t,i} = 2$ **do** ▷ Sellers update reservation price
  if $t - t_i^S \neq 0$ & $t - t_i^S \bmod \tau = 0$ **then** ▷ Every τ time steps after t_i^S
    $P_{t,i}^R = P_{t-1,i}^R \cdot \lambda$ ▷ Reduce by factor λ
  end if
end for
for $x = 1, \dots, L$ **do**
  $\{s_{t,i}, P_{t,i}^R, Y_{t,i}\}_{i \text{ s.t. } x_{t,i}=x}, P_{t,x} =$

Continuous Double Auction (Algorithm S2)$(\nu, \{s_{t,i}, P_{t,i}^R, Y_{t,i}\}_{i \text{ s.t. } x_{t,i}=x}, P_{t-1,x})$

end for

Algorithm S2 Continuous double auction in the original ABM.

Input: Parameter ν, Agent variables $\{s_{t,i}, P^R_{t,i}, Y_{t,i}\}_{i \text{ s.t. } x_{t,i}=x}$, location variable $P_{t-1,x}$

Output: Agent variables $\{s_{t,i}, P^R_{t,i}, Y_{t,i}\}_{i \text{ s.t. } x_{t,i}=x}$, location variable $P_{t,x}$

$\mathcal{B}_{t,x} = \{i \text{ s.t. } s_{t,i} = 0\}$ ▷ Buyers
$\mathcal{S}_{t,x} = \{i \text{ s.t. } s_{t,i} = 2\}$ ▷ Sellers
if $\mathcal{B}_{t,x} = \emptyset$ or $\mathcal{S}_{t,x} = \emptyset$ or $\max_{i \in \mathcal{B}_{t,x}} P^R_{t,i} < \min_{i \in \mathcal{S}_{t,x}} P^R_{t,i}$ **then**
    $P_{t,x} = P_{t-1,x}$ ▷ No transactions, so no update
else
    $\mathcal{O}^B_{t,x}, \mathcal{O}^S_{t,x} = \emptyset$ ▷ Sets of buyers and sellers in the order book
    $\mathcal{P}_{t,x} = \emptyset$ ▷ Set containing the prices of individual transactions
    for $i \sim \text{Uniform}\,(\mathcal{B}_{t,x} \cup \mathcal{S}_{t,x})$ **do** ▷ Draw uniformly at random without replacement
        if $i \in \mathcal{B}_{t,x}$ **then**
            $\mathcal{O}^B_{t,x} \leftarrow i$
        else
            $\mathcal{O}^S_{t,x} \leftarrow i$
        end if
        if $\mathcal{O}^B_{t,x} \neq \emptyset$ and $\mathcal{O}^S_{t,x} \neq \emptyset$ and $\max_{\iota \in \mathcal{O}^B_{t,x}} P^R_{t,\iota} > \min_{\iota \in \mathcal{O}^S_{t,x}} P^R_{t,\iota}$ **then**
            $j = \text{argmax}_{\iota \in \mathcal{O}^B_{t,x}} P^R_{t,\iota}$
            $h = \text{argmin}_{\iota \in \mathcal{O}^S_{t,x}} P^R_{t,\iota}$
            Remove j, h from $\mathcal{O}^B_{t,x}, \mathcal{O}^S_{t,x}$ respectively
            $\mathcal{P}_{t,x} \leftarrow \nu P^R_{t,j} + (1-\nu) P^R_{t,h}$
            $s_{t,h} = 1, Y_{t,h} = Y_{t,j}$ ▷ Agent j replaces agent h and becomes housed
        end if
    end for
    $P_{t,x} = \text{mean}(\mathcal{P}_{t,x})$
end if

S1.2 Detailed description of the learnable ABM

This section gives a more detailed description of the learnable ABM than Section A in Materials & Methods, and details the interpretation for each equation of the model. Table S2 can be used as a reference for notation throughout this section (although some notation overlaps with that of Table S1, there are a few differences and so we prefer to present the two tables as separate).

S1.2.1 General set-up

Agents are divided into K income classes, each characterized by income Y_k, $k = 1, \ldots, K$. All agents within the same income class, also named k-agents, are assumed to be identical and indistinguishable. The city is composed of L locations denoted by x.

S1.2.2 Demand

Let $M_{t,x,k}$ be the number of inhabitants of class k living at location x at time t. As shown in Table S2, this number is a real rather than an integer. We make this choice for computational reasons but, as we typically deal with large values of M, it does not substantially affect our results. We assume that each location x is characterized by an attractiveness $A_{t,x}$ that can change over time, given by

$$A_{t,x} = A^I_x \frac{\left(\sum_k M_{t-1,x,k} Y_k\right)}{\sum_x \left(\sum_k M_{t-1,x,k} Y_k\right)}. \tag{M1}$$

In the equation above, the first term A^I_x is an intrinsic attractiveness that is fixed over the simulation. It captures relatively permanent city features, such as amenities, schools, and public transport. The other term captures an attractiveness towards wealthier neighborhoods that can vary in time. It is defined by the mean, one-period lagged, income at location x, $\overline{Y}_{t-1,x} = \sum_k M_{t-1,x,k} Y_k / N$, divided by the mean income over the city $\overline{Y}_{t-1} = \sum_x \sum_k M_{t-1,x,k} Y_k / NL$. Thus, location x whose mean income is higher than average, i.e., $\overline{Y}_{t-1,x} > \overline{Y}_{t-1}$, is, *ceteris paribus*, more attractive than locations whose mean income is lower than average.

In their decision to move to location x, agents in income class k also take into account the affordability of location x, i.e., the difference between their willingness to pay, here simply captured by their income Y_k (in reality, willingness to pay is proportional to income, but, since we are not working with real-world data, for simplicity we take it equal to income) and the average price at $t-1$, $P_{t-1,x}$. The utility of k-agents for location x is given by an indirect utility function, derived from a standard utility function in urban economics and from the saturation of the budget constraint [1]. This reads:

$$V_{t,x,k} = \begin{cases} \left(Y_k - P_{t-1,x}\right)^{1-\beta} A^{\beta}_{t,x}, & Y_k > P_{t-1,x}, \\ 0, & Y_k \leq P_{t-1,x}. \end{cases} \tag{1}$$

where $\beta \in (0,1)$ gives the relative weight of attractiveness relative to affordability. When β is close to 1, agents care little about affordability, while when β is close to 0 the opposite holds. If location x is unaffordable ($Y_k \leq P_{t-1,x}$) then $V_{t,x,k} = 0$. Buyers in income class k are willing to bid up to their income Y_k, i.e., their reservation price $P^B_{t,x,k}$ is equal to Y_k.

Summing up, k-agents looking to buy a house in the city evaluate a utility $V_{t,x,k}$ for all locations x. They then choose a location x where they try to buy a house with probability $\pi_{t,x,k}$ proportional to $V_{t,x,k}$, i.e.

$$\pi_{t,x,k} = \frac{\left(\min\left(0, Y_k - P_{t-1,x}\right)\right)^{1-\beta} A^{\beta}_{t,x}}{\sum_x \left[\left(\min\left(0, Y_k - P_{t-1,x}\right)\right)^{1-\beta} A^{\beta}_{t,x}\right]}. \tag{M2}$$

We assume that a total of Q agents come to the city at each time step looking to buy a house, and that a share Γ_k of these agents is in income class k. The number of buyers of income class k at location x at time t, $N^B_{t,x,k}$ is given by

$$N^B_{t,x,k} = Q \Gamma_k \pi_{t,x,k}, \tag{M3}$$

Table S2: Notation for the learnable ABM.

Symbol	Set	Meaning
K	$\mathbb{N}$	Number of income classes
L	$\mathbb{N}$	Number of locations
N	$\mathbb{N}$	Number of houses per location
β	$\mathbb{R}[0,1]$	Preference for attractiveness relative to affordability
Q	$\mathbb{N}$	Total number of buyers at any time step
α	$\mathbb{R}[0,1]$	Probability to put house on sale
δ	$\mathbb{R}[0,1]$	Maximum reduction in seller reservation price
ν	$\mathbb{R}[0,1]$	Bargaining power of sellers
A^I	$\mathbb{R}^L$	Intrinsic attractiveness
Y	$\mathbb{R}^K$	Income
Γ	$\mathbb{R}^K$	Fraction of buyers by income class
M_t	$\mathbb{R}^{L\times K}$	Number of housed agents
A_t	$\mathbb{R}^L$	Total attractiveness
P_t	$\mathbb{R}^L$	Transaction price
π_t	$\mathbb{R}^{L\times K}[0,1]$	Probability to choose a location
N_t^B	$\mathbb{R}^{L\times K}$	Number of potential buyers
R_t	$\mathbb{R}^L$	Inventory of properties on sale
N_t^S	$\mathbb{R}^L$	Number of potential sellers
P_t^S	$\mathbb{R}^L$	Reservation price for sellers
D_t	$\mathbb{N}^L$	Number of transactions (Deals) that actually take place
π_t^D	$\mathbb{R}^{L\times K}[0,1]$	Probability that an agent is selected among the buyers to conclude a deal
D_t^B	$\mathbb{N}^{L\times K}$	Number of potential buyers that complete a transaction
D_t^S	$\mathbb{R}^{L\times K}$	Number of potential sellers that complete a transaction

We first indicate the parameters K and L that determine the size of the variables, next we indicate model-wide parameters (i.e., scalar quantities that are fixed in time) and location- or income class-specific parameters (size L or size K), and finally variables that can be location-specific (size L) or location-income-specific (size $L \times K$). We further show which quantities are constrained to the unit interval $[0, 1]$.

i.e., it is the expected value of a multinomial with $Q\Gamma_k$ trials and a probability vector given by the L values of $\pi_{t,x,k}$, for all locations x ((M3)).

S1.2.3 Supply

In each location x, the other side of the market is composed by sellers. We do not distinguish the income class of potential sellers, in the sense that we just keep track of the total number of agents willing to sell their house at location x at time t, $N^S_{t,x}$. The total number of sellers is obtained by summing the number of agents who wanted to sell at the previous time steps but did not succeed, $R_{t-1,x}$, and the number of agents who decide to put their house on sale at t. In turn, this number is given by a fixed fraction α of the agents that had not decided to sell before t, which represent the difference between the total number of agents residing at location x, N_x, and $R_{t-1,x}$. In formula,

$$N^S_{t,x} = R_{t-1,x} + \alpha(N_x - R_{t-1,x}). \tag{M4}$$

The way sellers determine their reservation price, i.e., the minimum price they are willing to accept, is more sophisticated. Here, we assume that sellers are not willing to accept any price below the average price at the previous time step, $P_{t-1,x}$, as long as there are more buyers than sellers. This choice captures the idea that, in this situation (known as a “sellers market”), sellers have more bargaining power than buyers. Conversely, when there are more sellers than buyers, sellers compete for the few buyers by being aggressive in reducing their reservation price. So, they are willing to accept offers that can be below $P_{t-1,x}$. We let $\phi_{t,x} = \left(\sum_k N^B_{t,x,k}\right)/N^S_{t,x}$ denote the ratio between the number of potential buyers and of potential sellers

at location x and time t. We then assume that sellers are willing to accept prices lower than $P_{t-1,x}$ by a fraction δ if there are more sellers than buyers, i.e., $\phi_{t,x} \to 0$. Conversely, when there are more buyers than sellers ($\phi_{t,x} \to \infty$), sellers are not willing to go below $P_{t-1,x}$. We interpolate between these extreme values of ϕ by assuming a hyperbolic tangent functional form, i.e., we assume that the sellers' reservation price $P^S_{t,x}$ is given by

$$P^S_{t,x} = P_{t-1,x}(1 - \delta(1 - \tanh((\sum_k N^B_{t,x,k})/N^S_{t,x}))) \tag{M5}$$

Here, we assume that sellers decide on their reservation price independently of their income class.

S1.2.4 Matching

At this point, the two sides of the market have been completely characterized, as we know the number of buyers in each income class $N^B_{t,x,k}$, their reservation price $P^B_{t,x,k}$, the number of sellers $N^S_{t,x}$, and their reservation price $P^S_{t,x}$. It remains to be determined how buyers and sellers are matched,t and how this matching impacts future prices and the social composition of neighborhoods.

To start, let $D_{t,x}$ denote the number of *deals* that occur at location x and time t, i.e., the number of transactions that effectively occur between buyers and sellers. This number is given by the "short side of the market", i.e., by the minimum between the number of potential buyers and potential sellers:

$$D_{t,x} = \min\left(\sum_k N^B_{t,x,k}, N^S_{t,x}\right) \tag{M6}$$

In case there are fewer deals than potential buyers, i.e., $D_{t,x} < \sum_k N^B_{t,x,k}$, we need to decide which potential buyers are successful in actually buying a house and which are not.

To do so, we assume that demand is satisfied on a pro-rata basis, although correcting the pro-rata assumption by making richer buyers more likely to secure a deal. This assumption captures a bargaining process in a more tractable way than explicitly simulating an auction. Thus, the probability that k-agents are able to secure a deal at location x and time t, denoted as $\pi^D_{t,x,k}$, is proportional to the number of potential buyers in that class, $N^B_{t,x,k}$, multiplied by the difference between the reservation price of k-buyers and that of sellers, $Y_k - P^S_{t,x}$:

$$\pi^D_{t,x,k} = \frac{N^B_{t,x,k} \cdot \left(Y_k - P^S_{t,x}\right)}{\sum_{k'}\left(N^B_{t,x,k'} \cdot \left(Y_{k'} - P^S_{t,x}\right)\right)}. \tag{M7}$$

Then, we compute the number of actual buyers of class k at time t in location x by a multinomial with $D_{t,x}$ trials and a parameter vector of length k given by $\pi^D_{t,x,k}$:

$$D^B_{t,x,k} = \text{multinomial}\left(D_{t,x}, \{\pi^D_{t,x,k}\}_k\right) \tag{M8}$$

We further compute the number of actual sellers $D^S_{t,x,k}$ by assuming that all agents living in location x are equally likely to sell, and so the share of k-agents among the sellers is proportional to the share of k-agents among the inhabitants, $M_{t-1,x,k}/N$:

$$D^S_{t,x,k} = D_{t,x}\frac{M_{t-1,x,k}}{\sum'_k M_{t-1,x,k'}} \tag{M9}$$

As above, this simplification ensures tractability, as keeping track of the number of sellers in each class over time would substantially increase the dimensionality of the space of state variables.

Next, after we determine which buyers and which sellers are successful in securing a deal, we need to determine the price of the transactions. First, we compute the average buyer reservation price $P^B_{t,x}$ as

$$P^B_{t,x} = \sum_k (Y_k D^B_{t,x,k} / \sum_{k'} D^B_{t,x,k'}). \tag{M10}$$

Then, we assume that the average transaction price is a weighted average between the reservation price of buyers and that of sellers:

$$P_{t,x} = \nu P^B_{t,x} + (1-\nu) P^S_{t,x} \quad \text{(M11)}$$

Here, ν denotes the bargaining power of sellers as, the larger ν, the higher the transaction price will be. Note that the transaction price is always in between the buyer and seller prices because it is always $P^B_{t,x} > P^S_{t,x}$. Indeed, (i) $P^B_{t,x} > P_{t-1,x}$ (as only buyers whose reservation price is larger than $P_{t-1,x}$ come to location x, see Equation (1)) and (ii) $P^S_{t,x} < P_{t-1,x}$, see Equation (M5).

S1.2.5 Update of state variables

It only remains to update the stocks of inhabitants $M_{t,x,k}$ of each class k in each location x and of unsuccessful sellers $R_{t,x}$ in x. For each class and each location, the number of inhabitants is given by the number of inhabitants on the previous time step, plus the buyers who secured a deal, minus the sellers who were able to sell:

$$M_{t,x,k} = \max\left(0, M_{t-1,x,k} + D^B_{t,x,k} - D^S_{t,x,k}\right). \quad \text{(M12)}$$

The number of unsuccessful sellers is obtained by summing the unsuccessful sellers on the previous time step to the number of sellers at t, subtracting the number of deals:

$$R_{t,x} = R_{t-1,x} + N^S_{t,x} - D_{t,x} \quad \text{(M13)}$$

S1.3 Comparison between the original ABM and the learnable ABM

We need to perform several modifications in order to make the original ABM learnable (see Table S3 for a summary).

As a general principle, while in the original ABM the state of the system was described by the variables of individual agents i, in the learnable ABM we only consider counts of how many agents are within each income class. For instance, in the original ABM we keep track of the state $s_{t,i}$, income $Y_{t,i}$ and location $x_{t,i}$ of each agent i, while in the learnable ABM the variable $M_{t,x,k}$ counts how many agents of income k are either housed or sellers at location x, the variable $R_{t,x,k}$ counts the sellers that were not successful in selling, and so on. This general modification does not lose much information. Indeed, the key heterogeneity that distinguishes agents in the original ABM is which income class they belong to, which is kept in the learnable ABM. We discuss below some examples where considering agent counts makes (or does not make) some difference. The rationale for this general principle is that we assume that we only observe aggregate data at the level of locations, and so we must be parsimonious with unobserved variables.

We now discuss the modifications one by one. First note that the probability for buyers to search for a home in a given location does not change from the original to the learnable ABM. For instance, although the specifications look different, Equation (M1) and lines 1 to 9 in Algorithm S1 are identical. At the same time, Equation (M2) is just a shorthand for lines 10 to 14.

When it comes to choosing a specific location, the learnable ABM essentially assumes the expected value of the stochastic process used in the original ABM. In the original ABM, individual agents belonging to a given income class k select a given location x by drawing from a categorical distribution—a multinomial distribution with one trial (line 20). Because all buyers belonging to the same income class are identical and have the same probability to choose a given location, it is completely equivalent to consider a multinomial distribution with γ_k trials. Indeed, this is what the learnable ABM does, except it considers the expected value of this distribution (Equation (M3)). This choice is, once again, to limit the amount of stochasticity: as the model does not observe potential buyers, it would have to estimate the realization of this variable, and this may create computational problems.

There is a similar difference in the computation of the number of sellers. The original ABM simulates the decisions of sellers individually (lines 23 to 29). As soon as a given housed agent becomes a seller with probability α, its state changes and that specific agent, from that point on, acts as a seller. In the learnable ABM we treat sellers as undistinguishable, and so it is sufficient to compute new sellers by drawing from a

Table S3: Comparison between original and learnable ABM. The first column reports the equation number of the learnable ABM (Table 1). The second column reports the lines of code that perform the same operation in the original ABM (Algorithm S1). The third column explains the changes. For each change, we indicate whether it fulfills principles P1 and P2.

Learnable	Original	Changes
All	All	Do not track individual agents, only consider agent counts (P1)
M1	lines 1 to 9	Identical
M2	lines 10 to 14	Identical
M3	lines 15 to 22	Instead of repeated draws from categorical distribution, use expected value of multinomial distribution (P1, P2)
M4	lines 23 to 29	Instead of repeated draws from Bernoulli distribution, use expected value of binomial distribution (P1, P2)
M5	line 27, lines 30 to 34	• Do not track the prices asked by individual agents, assume instead a location-specific seller reservation price (P1).<br>• Make the reservation price depend on the ratio between buyers and sellers, rather than discretely reducing it as sale is unsuccessful (P2).
M6-M13	lines 35 to 37	• Remove random ordering of buyers and sellers that is not observed (P1).<br>• Remove argmax operations (P2).

binomial distribution (in fact, by taking the expected value of that distribution, as in Equation (M4), again to limit the amount of stochasticity).

There is a more substantial difference in the way sellers determine their reservation price. In the original ABM they follow an *aspiration level heuristic* [2], i.e., sellers start from a markup on the market price (line 27), and then they decrease their reservation price if they are unable to sell (lines 30 to 34). The outcome of this heuristic is that prices tend to be higher in locations with higher demand, as sellers do not need to decrease their initial price much. We implicitly capture this dynamics in the specification of Equation (M5), as discussed in Section S1.2.3. However, according to our specification, all sellers in the same location have the same reservation price. This choice allows to treat sellers as undistinguishable in the learnable ABM, differently from the original ABM.

The final main difference lies in the way buyers and sellers are matched. Lacking information on individual transactions, we cannot write a computationally tractable likelihood by keeping the explicit representation of a continuous double auction (lines 35 to 37). So we try to keep its main features while using a more tractable form. We achieve this by giving more probability to be matched to buyers with higher income (Equation (M7)) and by computing the market price as the weighted mean of the average buyer and seller price, as in the original ABM. Note that the model keeps some stochasticity in the way the matching process works in the learnable ABM. In particular, we observe the number of transactions, and the realization of the matching affects the evolution of the social composition at each location, which is a key property of the model we wish to preserve.

S2 Supplementary results

In this section we discuss some additional results that were not shown in the main text.

S2.1 Hyperparameter selection

The learning algorithm described in Section B has a number of hyperparameters that must be set. It is beyond the scope of this section to explore the performance of the learning algorithm for each combination of hyperparameters, so we only explain how we assign some hyperparameter values, and explicitly show the effect of two hyperparameters that we consider particularly important.

- Initial guess for M_0. The first expectation step of our expectation-maximization algorithm requires

an initial guess for M_0. We take a uniform M_0 (i.e. a situation in which there is the same number of inhabitants of each income class in each location) to which we add some random noise. The realization of this noise is the only difference between runs of the learning algorithm on the same trace (all the other steps are deterministic). We experimented with a few options for the variance of the noise, finding that it did not have substantial impact on the results, and so simply decided to draw the noise from a standard Gaussian pdf in logarithmic space.

- Expectation-Maximization (EM) parameters. The EM algorithm keeps iterating the expectation and maximization steps until some variables converge or up to a maximum number of steps. We use a 5% threshold to decide on convergence, and allow for a maximum of 100 steps. Moreover, in the maximization step the gradient descent algorithm requires a learning rate and a number of learning steps (within each EM cycle). We choose a learning rate of 0.001 and a maximum of 4 learning steps. Overall, in our explorations the results were not particularly sensitive to the EM parameters.

- Number of epochs. In our preliminary experiments we found that going beyond 3 epochs only marginally increased accuracy, and the marginal gain kept decreasing with the number of epochs. In light of this preliminary evidence, we fixed the number of epochs to 5.

- Parameter δ. The parameter δ in Equation (M5) has no counterpart in the original ABM, in which the reservation price was set by individual sellers following an aspiration level heuristic. Thus, one needs to choose a value of δ that makes time series generated by the learnable ABM as similar as possible to time series generated by the original ABM. We experimented with a few values, noting that $\delta = 0.06$ seemed to yield the most similar time series.

- Other model parameters. All other parameters of the learnable ABM have a counterpart in the original ABM, and so we select the same values for both. These are: $L = 5$, $K = 3$, $N = 1000$, $Q = 500$, $\alpha = 0.1$, $\nu = 0.1$, $\beta = 0.5$, $Y = [10, 50, 90]$, $\Gamma = [0.5, 0.4, 0.1]$.

This leaves two hyperparameters whose effect we want to explore.

- Number of D^B samples. As detailed in Section B of Materials & Methods, our EM algorithm only considers a subset of all possible values of the latent variable whose likelihood is estimated in the expectation step. If the number of samples is higher, we expect that the performance of the algorithm improves, but this also leads to increased computational cost. We expect that increasing the number of D^B samples has a similar effect as increasing the number of epochs. However, because the sampling from all possible values of D^B is an original contribution of this paper, it is interesting to explicitly explore how it affects the performance of the algorithm.

- Standard deviation of the noise on observables, σ_P and σ_D for ϵ_P and ϵ_D respectively. As detailed in Section B, P_t and D_t are deterministic functions of their ancestors, and so in order to obtain a likelihood we model their observed values as a noisy proxy of the deterministic values. How close the observed values must be to the deterministic values is governed by a variance σ: the higher σ, the more observed values can be far from model values. First, note that the scale of σ does not matter: if both σ_P and σ_D are multiplied by the same number, the log-likelihood only shifts by a constant. What matters is the relative value of σ_P with respect to σ_D. When σ_P is larger than σ_D, the learning algorithm should give more importance to D_t, while in the opposite case it should give more importance to P_t. To study this effect, we set $\sigma_P = 1$ without loss of generality, and vary σ_D.

Figure S1 shows the Pearson correlation coefficient $\rho(M, \hat{M})$ between the ground-truth M and the estimate $\hat{M}$ in the 10 traces that we use as a training set, for different choices of hyperparameters. We consider three values of the number of D^B samples, namely 16, 64, and 256, and three values of σ_D, namely 0.01, 1, and 100.

Increasing the number of D^B samples monotonically increases $\rho(M, \hat{M})$, but the biggest gain is from 16 to 64 samples. The situation when changing σ_D is less clear: except for the case of 16 D^B samples, there is little difference between $\sigma_D = 0.01$ and $\sigma_D = 1$, and a small degrade in performance when choosing $\sigma_D = 100$.

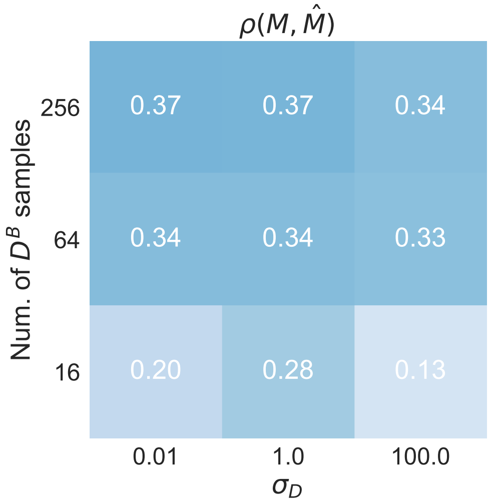

Figure S1: Hyperparameters selection for experiments with the original ABM, according to the Pearson correlation $\rho(M, \hat{M})$ between the ground truth M and estimated values $\hat{M}$. On the horizontal axis, we show the tested values for σ_D, which regulates the standard deviation of the prior distribution of the errors on the observed variable D. On the vertical axis, we show the tested values for the number of considered samples of the stochastic variable D^B (i.e. $|\Omega|$, described in Materials & Methods).

These results suggest that P_t and D_t carry similar information for estimating the latent variables, and so giving more importance to one variable over the other does not substantially change results (note that in our simulations P_t and D_t are on the same scale). For simplicity, we select $\sigma_D = 1$ for our experiments.

S2.2 Estimate of M_0 over time and epochs

Recall that, thanks to our online learning assumption, at each time step t the algorithm estimates M_0 while keeping all changes in the number of inhabitants in previous steps fixed. At the beginning of a new epoch, EM estimates M_0 starting from the latest estimate of M_0 in the previous epoch, and then repeats the same operations. A wildly changing estimate of M_0, both within the same epoch and across epochs, would indicate that the model cannot converge on an estimate.

Figure S2 considers the same simulation as the one shown in Figure 3. It shows that the estimate of M_0 is actually relatively stable, for most locations and income classes. For instance, in the case of locations $x = 0, 2, 3$ there are no noticeable trends and the algorithm remains stuck in a local minimum that does not correspond exactly to the ground truth (although it still has a good correlation). In locations $x = 1, 4$ there appears to be a trend that moves the estimation of M_0 closer to ground truth values (except for the case $x = 1, k = 0$), but convergence seems slow and, in our view, does not warrant increasing the number of epochs.

S2.3 Exploration of the loss

Here we explore in detail how the loss $\mathcal{L} = -\log \mathbb{P}(\mathbb{D}|M_0)$ depends on M_0 in three settings, shedding light into the performance of the algorithm.

We first consider the same simulation as the one shown in Figure 3 and in the section above, and focus on time step 1, i.e. we take as observables $\mathbb{D} = \{\tilde{P}_1, \tilde{D}_1\}$. Further taking D_1^B to be the same as the actual realization, we can explicitly compute the likelihood $\mathbb{P}(\mathbb{D}|M_0)$ and hence the loss $\mathcal{L}$ as the opposite of the log-likelihood for several possible values of the variable we want to estimate, M_0. In particular, we vary the number of inhabitants with highest income ($M_{0,x,k=2}$) at locations $x = 0$ and $x = 1$, ensuring that the total number of inhabitants at these locations remains equal to N. To do so, we fix the number of middle income inhabitants ($M_{0,x,k=1}$) to their ground truth value, and fix the number of low-income inhabitants ($M_{0,x,k=0}$)

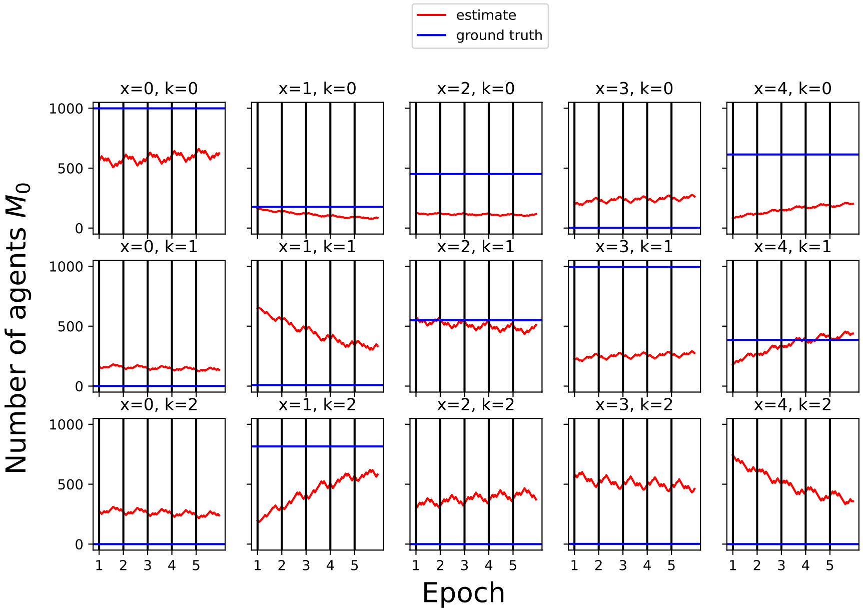

Figure S2: Estimate of $M_{0,x,k}$ over time and over multiple epochs, for the same simulation as the one shown in Figure 3. Each panel represents a value of location x and income class k.Each horizontal blue line is the ground truth value of M_0, each red line is the estimate of M_0 at a particular time step at a particular epoch. Black vertical lines distinguish the 5 epochs on which we run our experiments, within each epoch we estimate M_0 at each of 19 time steps.

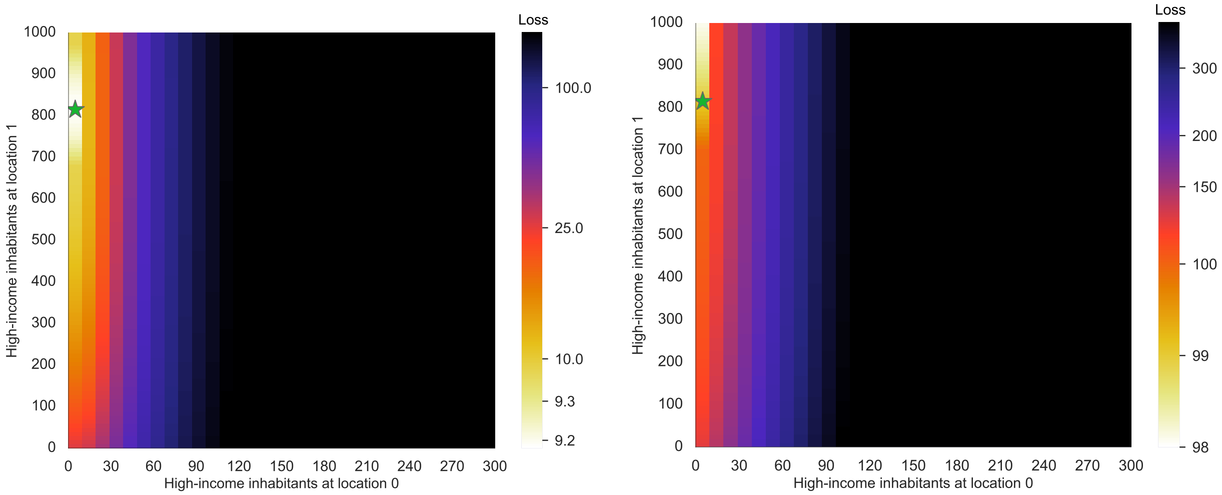

(a) Learnable ABM as ground truth

(b) Original ABM as ground truth

Figure S3: Heatmap of the loss (i.e., negative log likelihood) as a function of latent variables $M_{t=0,x=0,k=2}$ and $M_{t=0,x=1,k=2}$, representing the number of high-income inhabitants at two locations. The green star represents the ground truth value for these two variables. On the x axis we omit values beyond 300 since their loss is much larger, for better presentation.

to $M_{0,x,k=0} = N - M_{0,x,k=1} - M_{0,x,k=2}$. These constraints make it possible to vary $M_{0,x=0,k=2}$ between 0 and 999 and $M_{0,x=1,k=2}$ between 0 and 992. These values of M_0 include the ground truth, which corresponds to $M_{0,x=0,k=2} = 0$ and $M_{0,x=1,k=2} = 815$. (All other components of M_0 are the same as in the ground truth.)

Figure S3 shows the loss as a function of these values of M_0, both taking the learnable ABM and the original ABM as ground truth (for visualization purposes, we only show values of $M_{0,x=0,k=2}$ between

0 and 300, all other values lead to a much larger loss). In the case of the learnable ABM (shown on the left) there is no mis-specification, and the loss attains its minimum possible value. Since we take a Gaussian with unit variance and zero mean to model the error on P_t and D_t, the minimum value of $\mathcal{L}$ is $\mathcal{L} = -\log\left(\sum_{x=0}^{4} 1/\sqrt{2\pi}\exp(0)\right) - \log\left(\sum_{x=0}^{4} 1/\sqrt{2\pi}\exp(0)\right) = -9.2$ when the errors $P_t - \tilde{P}_t$ and $D_t - \tilde{D}_t$ are zero. As we see from the heatmap, the loss is well-behaved, in the sense that it does not display local minima and the minimum corresponds to the ground truth.

Interestingly, the gradient is much stronger when varying the number of high-income inhabitants at location 0, $M_{0,x=0,k=2}$, than when varying high-income inhabitants at location 1, $M_{0,x=1,k=2}$. This effect can be explained by the initial price (not shown) at location 1 being much higher than the initial price at location 0, so that only the highest income agents can afford location 1 in the first place. Therefore, making location 1 more or less attractive by increasing or decreasing the number of high-income agents inhabiting it does not make much of a difference to the distribution of buyers, compared to changing the number of high-income agents at location 0, which all the population can afford.

The right panel of Figure S3 takes the original ABM as the ground truth. The heatmap is not much different, thus suggesting that there is no major misspecification. However, the global minimum is a corner solution, at 1000 high-income inhabitants at location 1 and 0 high-income inhabitants at location 0, in contrast with the ground truth (the ground truth is the same for the original and the learnable ABM). Thus, while correctly guessing that location 1 has a much higher number of high-income inhabitants than location 0, the algorithm would not yield a perfect estimate. This qualitative result is in line with the quantitative results that we show in Figure 2 of the main paper.

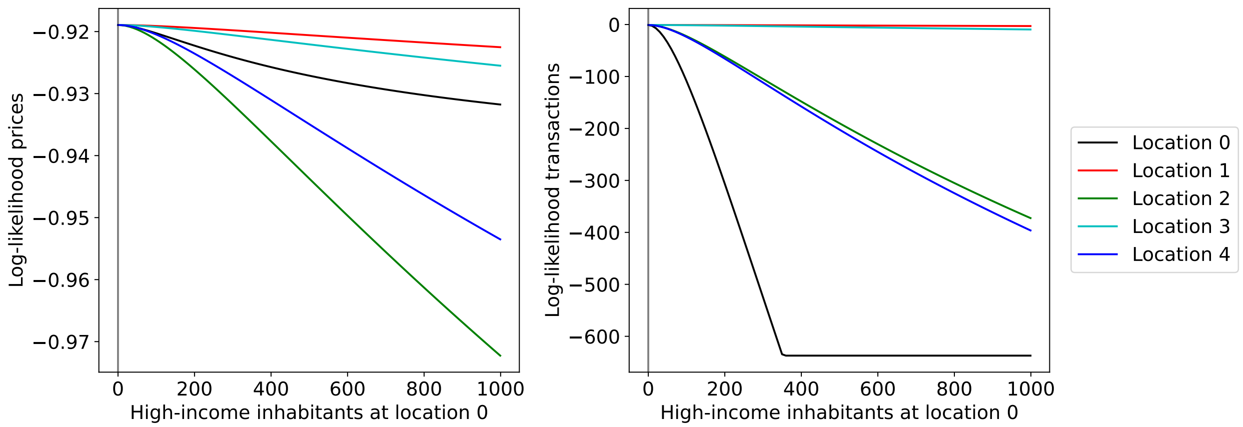

Figure S4: Log-likelihood of prices and transactions at all five locations as we vary the number of high-income inhabitants at location 0. Here, the learnable ABM is the ground truth, and the ground-truth number of high-income inhabitants is represented as a vertical grey line.

Next, Figure S4 shows the log-likelihood of prices $\log \mathbb{P}(\tilde{P}_t|M_0)$ and transactions $\log \mathbb{P}(\tilde{D}_t|M_0)$ as a function of the number of high-income inhabitants at location 0, $M_{0,x=0,k=2}$, when taking the ground-truth value of the $M_{0,x=0,k=2}$ (and the learnable ABM is the ground truth). Essentially, by taking the negative sum of all the components of the log-likelihood we obtain the loss, corresponding to a horizontal cut through the heatmap in Figure S3 (left) at the vertical coordinate of the ground truth. The advantage of the representation in Figure S4 is that we can understand how each component contributes to the loss, and whether there are some non-linearities that give insights into difficulties to estimate the latent variables.

First, the log-likelihood varies much more with D_t than with P_t. As an intuitive justification for why this is the case, consider the graphical model in Figure 5. In that graphical representation, P_t is influenced by M_0 only through several intermediate steps, while D_t is more directly influenced. In particular, changing the attractiveness of location 0 relative to the others changes demand across locations by a large margin, leading to very different values of D_t at each location. However, to propagate these differences to prices (at the same

time step!) we go through P_t^S, which only varies by a factor $\delta = 0.0625$ from the previous price, and so is not as sensitive to changes in M_0.

Second, the maximum of the log-likelihood is correctly achieved at $M_{0,x=0,k=2} = 0$, and then all components of the log-likelihood monotonically decrease with $M_{0,x=0,k=2}$. Focusing on the transactions (which dominate the loss), we see that the component of the likelihood corresponding to location $x = 0$ is the most affected, the likelihood at locations 2 and 4 is also strongly affected, while the likelihood at 1 and 3 is barely affected. The reason is that buyers at locations 1 and 3 are almost exclusively high-income, whereas locations 2 and 4 have many middle-income buyers (as location 0 does). So, increasing the number of high-income buyers at location 0 strongly decreases the number of middle-income buyers at 2 and 4 (in a sense, these three locations get in competition), but it does not have a strong effect on locations 1 and 3.

Third, it is interesting that the log-likelihood for transactions at location 0 flattens out after a number of high-income inhabitatns $M_{0,x=0,k=2} = 350$. This effect is due to a supply constraint: increasing the number of high-income inhabitants at location 0 substantially increases demand, but the number of sellers is fixed, so, when the number of buyers becomes larger than the number of sellers, the number of transactions remains fixed (Equation (M6)). Increasing the number of buyers still puts upward pressure on prices, and indeed the log-likelihood of prices does not flatten out.

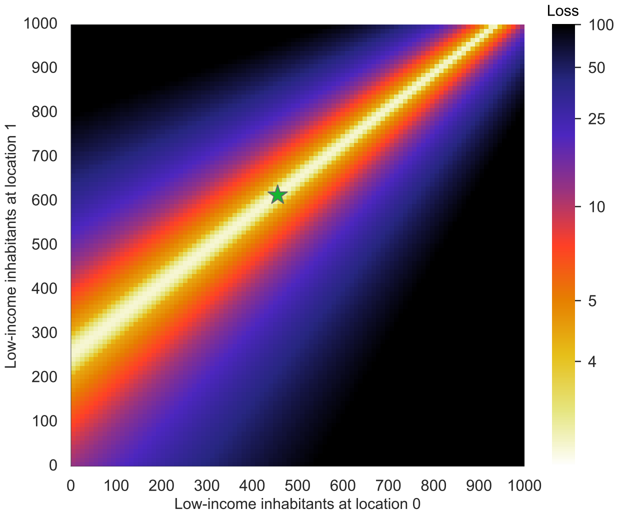

Figure S5: Heatmap of the loss (i.e., negative log likelihood) as a function of latent variables $M_{t=0,x=0,k=0}$ and $M_{t=0,x=1,k=0}$, representing the number of low-income inhabitants at the two locations of this model. The green star represents the ground truth value for these two variables.

As a last example, we consider a simplified setting with $L = 2$ locations, which allows to visualize a higher portion of the latent variable space than with $L = 5$ locations. Indeed, with $L = 5$ locations we have 10 degrees of freedom in M_0 (considering $K = 3$ and the constraint that the total number of inhabitants is N at each location), but we can only visualize how the loss changes by varying 2 components and keeping all others fixed. Instead, with $L = 2$ locations we only have 4 degrees of freedom, so varying 2 components of M_0 at a time gives a more complete picture.

For simplicity, we initialize the model with the same parameters as above, focusing on the locations 2 and 4 above. In this case, we have $M_{x=0,k,t=0} = [451, 549, 0]$ and $M_{x=1,k,t=0} = [614, 386, 0]$ as ground truth. This time, we vary the number of low-income inhabitants $M_{0,x,k=1}$ at both locations, again using the same method as above to ensure that the total number of inhabitants is always N at each location.

The results of this analysis are shown in Figure S5. Differently from Figure S3, here the minimum of the loss is not attained at a single combination of values of M_0. Instead, all points on a line that crosses the latent variable space from $M_{x=0,0,0}, M_{x=1,0,0} = [0, 250]$ to $[900, 1000]$ lead to a value very close to the minimum loss (with only two locations, this value is $\mathcal{L} = -\log\left(\sum_{x=0}^{1} 1/\sqrt{2\pi}\exp(0)\right) - \log\left(\sum_{x=0}^{1} 1/\sqrt{2\pi}\exp(0)\right) = -3.7$).

Intuitively, with just two locations, what matters is the relative attractiveness at one location compared to the other location. So, as long as there are fewer low-income inhabitants at one location than at the other location, several possible configurations of M_0 lead to very similar values for the loss.

This situation constitutes an *identification problem*: the model is not able to identify the ground truth, and any inference algorithm could converge on any value on the white line Figure S5. We conjecture that similar issues could prevent the learning algorithm from obtaining a perfect estimate for M. Note that this problem is not due to the translation into a learnable form, but intrinsic to the ABM under scrutiny: many possible configurations of agents could lead to the same observable outcome. Our approach allows to formally define and diagnose such issues, thus allowing ABM researchers to take into account the learnability of their model from observed data.

References

[1] Marco Pangallo, Jean-Pierre Nadal, and Annick Vignes. Residential income segregation: A behavioral model of the housing market. *Journal of Economic Behavior & Organization*, 159:15–35, 2019.

[2] Florian M Artinger and Gerd Gigerenzer. Heuristic pricing in an uncertain market: Ecological and constructivist rationality. *Available at SSRN 2938702*, 2016.
